# Supplementary material for: Comparative transcriptome analysis of the cold resistance of the sterile rice line 33S
Source: PLoS One. 2022 Jan 14;17(1):e0261822. doi: 10.1371/journal.pone.0261822 (PMC8759683; doi:10.1371/journal.pone.0261822)
Supplement: S1 Table — (DOC) [file pone.0261822.s001.doc]

**Table S1 P**rimers used for qRT-PCR in this study

| **Gene locus** | **Primer** | **sequence（5'-3')** |
| --- | --- | --- |
| Os01g0124000 | Os01g0124000-F | CGAAACCGATGGAAACAAA |
|  | Os01g0124000-R | GGCGCACTGCTTCACCT |
| Os01g0764900 | Os01g0764900-F | TACAAGCGTGCTGTCCTCA |
|  | Os01g0764900-R | AGTTGCCACTGCATTAGGG |
| Os01g0971800 | Os01g0971800-F | CAGCAGCAGCAAGAGGG |
|  | Os01g0971800-R | GGAGGCGAAGATGTGGTC |
| Os02g0685200 | Os02g0685200-F | ACCGAGCATCCCGACTG |
|  | Os02g0685200-R | CCGAATAGCCTGAAGACTGA |
| Os03g0161900 | Os03g0161900-F | GCGGAGGTTGTGAAACTAAG |
|  | Os03g0161900-R | GGGTTGAGAAATGGCACTATG |
| Os03g0293000 | Os03g0293000-F | CAGTCCGCCTTCCTGTCCAC |
|  | Os03g0293000-R | CAGCCTCTTCCATAGTCACCTCAT |
| Os06g0474800 | Os06g0474800-F | CGGCAGCAACGCTACAATAA |
|  | Os06g0474800-R | CACCAGGTTTCGTCCAGTCC |
| Os10g0509700 | Os10g0509700-F | AATCCTCGATCTGGTTGC |
|  | Os10g0509700-R | GAGATACGAATACGATGAACC |
| Actin | Actin-F | AAGTGCGGCATCAACTACCAG |
|  | Actin-R | GATGTCGATGCGGGAGAACAC |
