# Supplementary material for: Comparative transcriptome analysis of the cold resistance of the sterile rice line 33S
Source: PLoS One. 2022 Jan 14;17(1):e0261822. doi: 10.1371/journal.pone.0261822 (PMC8759683; doi:10.1371/journal.pone.0261822)
Supplement: Attachment — Submitted filename: Response to Reviewers.docx [file pone.0261822.s013.docx]

PONE-D-21-12476
Comparative transcriptome analysis of the cold resistance of the sterile rice line 33S
PLOS ONE
Dear Dr. Zhu,
Thank you for submitting your manuscript to PLOS ONE. After careful consideration, we feel that it has merit but does not fully meet PLOS ONE’s publication criteria as it currently stands. Therefore, we invite you to submit a revised version of the manuscript that addresses the points raised during the review process.
Please submit your revised manuscript by Jul 26 2021 11:59PM. If you will need more time than this to complete your revisions, please reply to this message or contact the journal office at [plosone@plos.org](mailto:plosone@plos.org). When you're ready to submit your revision, log on to <https://www.editorialmanager.com/pone/> and select the 'Submissions Needing Revision' folder to locate your manuscript file.
Please include the following items when submitting your revised manuscript:

- A rebuttal letter that responds to each point raised by the academic editor and reviewer(s). You should upload this letter as a separate file labeled 'Response to Reviewers'.
- A marked-up copy of your manuscript that highlights changes made to the original version. You should upload this as a separate file labeled 'Revised Manuscript with Track Changes'.
- An unmarked version of your revised paper without tracked changes. You should upload this as a separate file labeled 'Manuscript'.

If you would like to make changes to your financial disclosure, please include your updated statement in your cover letter. Guidelines for resubmitting your figure files are available below the reviewer comments at the end of this letter.

If applicable, we recommend that you deposit your laboratory protocols in protocols.io to enhance the reproducibility of your results. Protocols.io assigns your protocol its own identifier (DOI) so that it can be cited independently in the future. For instructions see: <http://journals.plos.org/plosone/s/submission-guidelines#loc-laboratory-protocols>. Additionally, PLOS ONE offers an option for publishing peer-reviewed Lab Protocol articles, which describe protocols hosted on protocols.io. Read more information on sharing protocols at <https://plos.org/protocols?utm_medium=editorial-email&utm_source=authorletters&utm_campaign=protocols>.

We look forward to receiving your revised manuscript.

Kind regards,

Jian Zhang
Academic Editor
PLOS ONE
Journal Requirements:

When submitting your revision, we need you to address these additional requirements.

1. Please ensure that your manuscript meets PLOS ONE's style requirements, including those for file naming. The PLOS ONE style templates can be found at
<https://journals.plos.org/plosone/s/file?id=wjVg/PLOSOne_formatting_sample_main_body.pdf> and
<https://journals.plos.org/plosone/s/file?id=ba62/PLOSOne_formatting_sample_title_authors_affiliations.pdf>

Reply ：We have modified the style of PLoS One's style requirements.

2. We suggest you thoroughly copyedit your manuscript for language usage, spelling, and grammar. If you do not know anyone who can help you do this, you may wish to consider employing a professional scientific editing service.
Whilst you may use any professional scientific editing service of your choice, PLOS has partnered with both American Journal Experts (AJE) and Editage to provide discounted services to PLOS authors. Both organizations have experience helping authors meet PLOS guidelines and can provide language editing, translation, manuscript formatting, and figure formatting to ensure your manuscript meets our submission guidelines. To take advantage of our partnership with AJE, visit the AJE website (<http://learn.aje.com/plos/>) for a 15% discount off AJE services. To take advantage of our partnership with Editage, visit the Editage website ([www.editage.com](http://www.editage.com/)) and enter referral code PLOSEDIT for a 15% discount off Editage services.  If the PLOS editorial team finds any language issues in text that either AJE or Editage has edited, the service provider will re-edit the text for free.Upon resubmission, please provide the following:
The name of the colleague or the details of the professional service that edited your manuscript A copy of your manuscript showing your changes by either highlighting them or using track changes (uploaded as a *supporting information* file) . A clean copy of the edited manuscript (uploaded as the new *manuscript* file)”

Reply ：We have completed the revision in AJE.

3. We note that Figure 1 in your submission contain copyrighted images. All PLOS content is published under the Creative Commons Attribution License (CC BY 4.0), which means that the manuscript, images, and Supporting Information files will be freely available online, and any third party is permitted to access, download, copy, distribute, and use these materials in any way, even commercially, with proper attribution. For more information, see our copyright guidelines: <http://journals.plos.org/plosone/s/licenses-and-copyright>.

We require you to either (1) present written permission from the copyright holder to publish these figures specifically under the CC BY 4.0 license, or (2) remove the figures from your submission:

1. You may seek permission from the original copyright holder of Figure 1 to publish the content specifically under the CC BY 4.0 license.

We recommend that you contact the original copyright holder with the Content Permission Form (<http://journals.plos.org/plosone/s/file?id=7c09/content-permission-form.pdf>) and the following text:
“I request permission for the open-access journal PLOS ONE to publish XXX under the Creative Commons Attribution License (CCAL) CC BY 4.0 (<http://creativecommons.org/licenses/by/4.0/>). Please be aware that this license allows unrestricted use and distribution, even commercially, by third parties. Please reply and provide explicit written permission to publish XXX under a CC BY license and complete the attached form.”

Please upload the completed Content Permission Form or other proof of granted permissions as an "Other" file with your submission.

In the figure caption of the copyrighted figure, please include the following text: “Reprinted from [ref] under a CC BY license, with permission from [name of publisher], original copyright [original copyright year].

2. If you are unable to obtain permission from the original copyright holder to publish these figures under the CC BY 4.0 license or if the copyright holder’s requirements are incompatible with the CC BY 4.0 license, please either i) remove the figure or ii) supply a replacement figure that complies with the CC BY 4.0 license. Please check copyright information on all replacement figures and update the figure caption with source information. If applicable, please specify in the figure caption text when a figure is similar but not identical to the original image and is therefore for illustrative purposes only.

Reply ：Figure 1 without copyright protection. The pictures and data are obtained by the author in this paper experiment.

Additional Editor Comments (if provided):
please carefully address all the questions raised by both reviewers. In particular, the authors should provide more interpretation of the biological significance behind the RNA seq data. e.g. function-known genes involved in low-temperature response, which has been suggested by reviewer 2. The writing needs a thorough revision, as numerous typos, errors could be found in the text, while some unrelated contents should be excluded to make the logic flow clear.


[Note: HTML markup is below. Please do not edit.]

Reviewers' comments:

Reviewer's Responses to Questions

**Comments to the Author**

1. Is the manuscript technically sound, and do the data support the conclusions?

The manuscript must describe a technically sound piece of scientific research with data that supports the conclusions. Experiments must have been conducted rigorously, with appropriate controls, replication, and sample sizes. The conclusions must be drawn appropriately based on the data presented.

Reviewer #1: Yes

Reviewer #2: Partly

2. Has the statistical analysis been performed appropriately and rigorously?

Reviewer #1: Yes

Reviewer #2: I Don't Know

3. Have the authors made all data underlying the findings in their manuscript fully available?

The [PLOS Data policy](http://www.plosone.org/static/policies.action#sharing) requires authors to make all data underlying the findings described in their manuscript fully available without restriction, with rare exception (please refer to the Data Availability Statement in the manuscript PDF file). The data should be provided as part of the manuscript or its supporting information, or deposited to a public repository. For example, in addition to summary statistics, the data points behind means, medians and variance measures should be available. If there are restrictions on publicly sharing data—e.g. participant privacy or use of data from a third party—those must be specified.

Reviewer #1: No

Reviewer #2: Yes

4. Is the manuscript presented in an intelligible fashion and written in standard English?

PLOS ONE does not copyedit accepted manuscripts, so the language in submitted articles must be clear, correct, and unambiguous. Any typographical or grammatical errors should be corrected at revision, so please note any specific errors here.

Reviewer #1: No

Reviewer #2: No

5. Review Comments to the Author

Please use the space provided to explain your answers to the questions above. You may also include additional comments for the author, including concerns about dual publication, research ethics, or publication ethics. (Please upload your review as an attachment if it exceeds 20,000 characters)

Reviewer #1: The manuscript drafted by Xie et al. profiled transcriptome changes of cold-resistant rice variety 33S and cold-sensitive variety Nipponbare in response to 7-days’ cold stress at seedling stage. The comparative analyses identified DEGs and regulatory pathways distinguished between 33S and Nipponbare. The study proposed regulation mechanisms in response to cold stress of rice. However, the manuscript hasn’t met the standard for publication at this moment. A major revision is recommended.

Comments:
1. The abstract should be succinct. The very detailed information, such as the ones in parentheses, are suggested to be excluded from the abstract. Grammar issues found in Abs

Reply: we have simplified and modified the syntax of the abstract.

2. I didn’t see any Tables attached in the manuscript. Table 1, 3, and 4 were mentioned in paper (Table 2 is missing) but not shown.

Reply: the form has been placed in the appropriate position in the article.

3. Materials and Methods section needs more detailed descriptions. Such as the ‘RNA sequencing (RNA-seq) data analysis’ section: reference genome version needs to be clarified; No QC step or reads trimming? Line 122 needs citation; Line 124, which method used for p-value correction? What’s key parameters used in Tophat and HTseq?

Reply: Thank you for your suggestions. We have enriched the contents of materials and methods, and added corresponding references.

4. Line 92, the seedling for experiment is ‘fifty-one-week-old seedlings’? That does not make sense at all.

Reply: Sorry, this is a clerical error. We have replaced fifth one week old seeds with fifth one week old seeds.

5. Figures, figure legends, and Tables are very messy.
1). No figure legend for Figure 1.

Reply: the annotation of Figure 1 has been added.

2) current Figure 1 legend is actually for Figure 2. For Figure 2 legend, please add a sentence to clarify ‘N’ and ‘CK’. Line 149-153 is a better legend for figure 2 rather than the one in Line 185-186.

Reply: the description has been added as required and the drawing notes have been modified.

3) Supplementary tables should be consistent with their order appear in paper. Line174-179.

Reply: I'm sorry for our carelessness. Modified as required

4) Figure 3 C and D, what’s Q-vaule? No ‘red line’ in figure 3 as descripted in it's legend.
Figure 3A, what’s the asterisk indicated?

Reply: Q-value has been modified to p-value. The description of the red line has been deleted. The asterisk in Figure 3A has no meaning and has been removed.

5) Line 300-301, it should be Figure 2 instead of Figure 1.

Reply: sorry, it has been modified

6) Figure 5 legend is missing.

Reply: sorry, we have added a drawing note.

6. Line 145, inappropriate statement: ‘no 33S seedlings were affected by low temperature’. Since a large number DEGs were identified, there must be affections in 33S. You could say something like ‘no 33s seedlings died after cold treatment’.

Reply: Thank you for your suggestion. We have modified this part according to your suggestion

7. I didn’t understand the logic in Line 179-182.

Reply: Thank you for your suggestion. We have recounted this part.

8. Throughout the paper, please make sure gene names are in italic format but not for protein names.

Reply: we have checked and modified the format in the full text as required.

9. The RNAseq data should be deposit in public database.

Reply: we have submitted data in the public database and provided data acquisition channels in the paper.

10. Line 170-172, what’s Q <0.05 mean? And also ‘Differences in gene
expression in the six samples were examined using a threshold…’ is not accurate. DEGs are determined by two conditions not individual sample. Line 172-173 is a better description. Combine line 171-173.

Reply: Thank you for your comments. Q < 0.05 has been revised to P < 0.05. We re described the contents of lines 170-172.

Reviewer #2: The authors tried to investigate the low-temperature response mechanisms by comparative transcriptomic analysis between a TGMS variety (33S) and a cold-sensitive variety (Nipponbare). This study is meaningful for our understanding of low-temperature response mechanisms in rice seedling, as well as genetic improvement of cold-tolerant rice in future. However, the manuscript has to be improved in both science and writing for publication in PLOS ONE.

Major comments:
(1) No tables found in this manuscript.

Reply: we have put the picture in the right place according to the text.

(2) The analysis is insufficient. The authors performed RNA-Seq analysis, to focus on the biological question, they should focus on the specifically biological processes or DEGs in low-temperature response of 33S in contrast with Nipponbare.

Reply: Thank you for your suggestion. We added the analysis of differential genes in the manuscript.

(3) To be more convincing, the authors should also analyze the function-known genes involved in low-temperature response, not just common GO and KEGG analysis.

Reply: Thank you for your suggestion. We analyzed and discussed the known genes involved in hypothermia (such as) in the revised manuscript.

(4) Some descriptions were unrelated to the topic, and the logic is a little bit confusing. For example, the ARR9 is reported to be involved in cold treatment, but it is responsive to low-temperature treatment in the Nipponbare variety, which is a cold-sensitive variety. This cannot explain why 33S is cold-tolerant.

Reply: Thank you for your suggestion. However, the current research results show that the mechanism of plant low temperature tolerance is very complex, and many genes are involved. Although 33S material has stronger adaptability to low temperature than Nipponbare, there must be some common genes responding to low temperature stress at the same time.

(5) Several conclusions were over speculative from the RNA-seq data throughout the manuscript. For example, the line 250~252 is not supported by only expression of ARRs, which needs more evidences, such as enrichment of GO or KEGG or others. The authors should draw any conclusion throughout the manuscript, cautiously.

Reply: Thank you for your comments. The result data provided in our paper is indeed insufficient. We have modified the description of the conclusion。

Minor comments:

(1) The English writing need to be polished by a native English speaker or language service to correct spelling and grammar errors. For example, ‘Ehe’ should be ‘The’ in line 40. The first word of cold stress in line 48 should be capitalized. The gene names should be italicized in line 135, line 227~229, line 240~247, line 254, line 287~289, line 320~ 337, etc.

Reply: We apologize for our carelessness. We have carefully checked and revised the full manuscript.

(2) Figure legends and figures should be of good shape. Some figure legends were not detailed, such as the means of green and red dots in line 185 (Figure 2). There were no A, B, and C marked in Figure 1. In Figure 1A, the roots showed shorter than control, which is contradictory with the statistical result of Figure 1B. In Figure 1C, ‘33S-Cold vs 33S-Cold’ should be ‘33S-Cold vs 33S-CK’. The statistical test, such as student’s t-test, should be added in Figure 4. Letter numbers have brackets in Figure 5, which is not observed in other Figures.

Reply: Thank you for your comments. We have modified the picture format and annotation according to your comments.
(3) The section of ‘Illumina RNA-seq and assembly analyses’ is the most basic for RNA-seq analysis, and was uncorrelated to the topic. It should be deleted to make the manuscript more concise or move to “Materials and Methods” section.

Reply: Thank you for your suggestion. We have removed this part.

6. PLOS authors have the option to publish the peer review history of their article ([what does this mean?](https://journals.plos.org/plosone/s/editorial-and-peer-review-process#loc-peer-review-history)). If published, this will include your full peer review and any attached files.


If you choose “no”, your identity will remain anonymous but your review may still be made public.


**Do you want your identity to be public for this peer review?** For information about this choice, including consent withdrawal, please see our [Privacy Policy](https://www.plos.org/privacy-policy).

Reviewer #1: No

Reviewer #2: No

[NOTE: If reviewer comments were submitted as an attachment file, they will be attached to this email and accessible via the submission site. Please log into your account, locate the manuscript record, and check for the action link "View Attachments". If this link does not appear, there are no attachment files.]

While revising your submission, please upload your figure files to the Preflight Analysis and Conversion Engine (PACE) digital diagnostic tool, <https://pacev2.apexcovantage.com/>. PACE helps ensure that figures meet PLOS requirements. To use PACE, you must first register as a user. Registration is free. Then, login and navigate to the UPLOAD tab, where you will find detailed instructions on how to use the tool. If you encounter any issues or have any questions when using PACE, please email PLOS at [figures@plos.org](mailto:figures@plos.org). Please note that Supporting Information files do not need this step.
