# Supplementary material for: Comparative transcriptome analysis of the cold resistance of the sterile rice line 33S
Source: PLoS One. 2022 Jan 14;17(1):e0261822. doi: 10.1371/journal.pone.0261822 (PMC8759683; doi:10.1371/journal.pone.0261822)
Supplement: Attachment — Submitted filename: Minor Revision - Response to Reviewers.docx [file pone.0261822.s014.docx]

6. Review Comments to the Author

Please use the space provided to explain your answers to the questions above. You may also include additional comments for the author, including concerns about dual publication, research ethics, or publication ethics. (Please upload your review as an attachment if it exceeds 20,000 characters)

Reviewer #1: The revised version manuscript entitled with ‘Comparative transcriptome analysis of the cold resistance of the sterile rice line 33S’ was improved and basically addressed most of my concerns. However, a couple of points need to be clarified before acceptation. Especially, please specify criterion used for GO and KEGG enrichment analyses as some key conclusions were drawn from them (Point 2.2 in below).

1. In the abstract section, it uncommon to describe pathway by ‘reduced’ or ‘increased’. It can be replaced by ‘repressed’ or ‘enhanced’.

**Response:** Thanks for your suggestion. They were changed (line 31 and line 33, page 2).

2. Materials and Methods section:

1) please specify the reference genome version, such as v7 or something else, used for RNAseq analysis.

**Response:** the genome we used was GCF_000005425.2_Build_4.0_genomic, and we added it in the M&M.

2). There’s no criterion described for GO and KEGG pathway enrichment analyses. The E value <= 10-5 is expect value during BLAST but not for determining statistical significance for enrichment. I checked Table S8, S9, and S10 as the results for functional enrichment analyses and it seems most terms with p-value >0.05 which indicated statistically unsignificant. Since some conclusions were based on the functional enrichment analyses, please make it clear.

**Response:** Thanks for your suggestion, we had corrected these descriptions, only that p<0.05 were described as significant, and those p>0.05 were considered not to enriched. But we still thought the DEGs in such pathways were valuable to reveal the regulation mechanism of cold tolerance. Please see the line 157-164, page 7, the corrections were made.

3. Figure 1: the labels for ‘33S’ and ‘Nipponbare’ are wrong; no letters indicated significant difference between comparison, but mentioned in legend; The error bar in Figure1C looked inconsistent with Figure 1B, did you used standard error or standard deviation for error bar?

**Response:** we both used the standard deviation (SD), and this description was added into figure legends. Actually, the error bar in Figure1C looked inconsistent with Figure 1B, perhaps the root length were affected by cold leading to bigger difference.

4. Above Figure 2, on page 7, it should be ‘In contrast, more up-regulated genes were identified in Nipponbare variety’ instead of ‘down-regulated’.

**Response:** Thanks, it has been changed.

5. Figure 2 legend, ‘|log2FoldChange| >1’ is actually ‘|log2FoldChange| >=1’ to be consistant with Materials and Methods?

**Response:** Thanks, it has been changed.

6. Figure 3C and D, does the ‘qvalue’ in figure actually mean ‘pvalue’? What’s the ‘rich factor’ indicated?

**Response:** yes, qvalue is a adjust pvalue. And the rich factor means the ratio of DEGs in total specific annotated pathway/term.

Reviewer #2: (No Response)
